# The factors influencing clinician use of hypertension guidelines in different resource settings: a qualitative study investigating clinicians’ perspectives and experiences

**DOI:** 10.1186/s12913-021-06782-w

**Published:** 2021-08-03

**Authors:** Amelia Kataria Golestaneh, Jonathan M Clarke, Nicholas Appelbaum, Carmen Rodriguez Gonzalvez, Arun P Jose, Richu Philip, Neil R Poulter, Thomas Beaney

**Affiliations:** 1 Department of Primary Care and Public Health, Imperial College London, St Dunstan’s Road, W6 8RP London, UK; 2 Centre for Mathematics of Precision Healthcare, Department of Mathematics, Imperial College London, London, UK; 3 Institute of Global Health Innovation, Imperial College London, London, UK; 4 Centre for Chronic Conditions and Injuries, Public Health Foundation of India, New Delhi, India; 5 Imperial Clinical Trials Unit, School of Public Health, Imperial College London, London, UK

**Keywords:** Clinical guidelines, Hypertension, Guideline use

## Abstract

**Background:**

Hypertension accounts for the greatest burden of disease worldwide, yet hypertension awareness and control rates are suboptimal, especially within low- and middle-income countries. Guidelines can enable consistency of care and improve health outcomes. A small body of studies investigating clinicians’ perceptions and implementation of hypertension guidelines exists, mostly focussed on higher income settings. This study aims to explore how hypertension guidelines are used by clinicians across different resource settings, and the factors influencing their use.

**Methods:**

A qualitative approach was employed using convenience sampling and in-depth semi-structured interviews. Seventeen medical doctors were interviewed over video or telephone call from March to August 2020. Two clinicians worked in low-income countries, ten in middle-income countries, and five in high-income countries. Interviews were recorded, transcribed, and coded inductively. Reflexive thematic analysis was used.

**Results:**

Themes were generated at three levels at which clinicians perceived influencing factors to be operating: healthcare worker, healthcare worker interactions with patients, and the wider health system. Within each level, influencing factors were described as barriers to and facilitators of guideline use. Variation in factors occurred across income settings. At the healthcare worker level, usability of guidelines, trust in guidelines, attitudes and views about guidelines’ purpose, and relevance to patient populations were identified as themes. Influencing factors at the health system level were accessibility of equipment and medications, workforce, and access to healthcare settings. Influences at the patient level were clinician perceived patient motivation and health literacy, and access to, and cost of treatment, although these represented doctors’ perceptions rather than patient perceived factors.

**Conclusions:**

This study adds a high level global view to previous studies investigating clinician perspectives on hypertension guideline use. Guidelines should be evidence-based, regularly updated and attention should be given to increasing applicability to LMICs and a range of healthcare professionals.

**Supplementary Information:**

The online version contains supplementary material available at 10.1186/s12913-021-06782-w.

## Background

Hypertension accounts for the greatest burden of disease worldwide [1–3]. Its rising prevalence has been well documented and is predicted to surpass 1.56 billion by 2025 [4, 5]. The global significance of this trend lies in hypertension’s role as a leading modifiable risk factor for cardiovascular disease, including ischaemic heart disease and stroke, and chronic kidney disease (CKD) [4, 5]. In 2015, complications of hypertension accounted for an estimated 10.7 million deaths [6]. Approximately 90 % of the cardiovascular disease burden worldwide is held by low- and middle-income countries (LMICs), which have seen an absolute increase in hypertension prevalence of 7.7 % from 2000 to 2010, affecting 1 billion people in LMICs in 2010 [4, 5, 7, 8]. The reasons behind the rapid epidemiological transition in LMICs have been attributed to growing population size, ageing populations and fundamental changes in environmental risk factors and behaviour, such as unhealthy diets, alcohol and physical inactivity, often owing to urbanisation and Westernisation [3, 4]. The subsequent dual burden of communicable and non-communicable diseases is compounded by the trend of cardiovascular disease onset at an earlier age in LMICs, creating a disease burden inadequately managed by weaker health systems [4, 5, 9, 10].

Despite its growing burden, hypertension awareness and control rates remain suboptimal worldwide, particularly among economically developing countries [4, 8]. The 2018 May Measurement Month (MMM) study which screened 1.5 million people worldwide, of whom 87.8 % were from LMICs, found lower treatment and control rates in low-income countries (LICs) in particular [11]. Mills et al. identified that globally, 46.5 % of hypertensive adults in 2010 were aware of their condition, of whom 36.9 % were taking antihypertensive medication and a meagre 13.8 % were regarded as having controlled blood pressure (BP) [4]. Patients in high-income countries (HICs) were found to have almost double the awareness and treatment rates of those in LMICs, and there was a four-fold difference in the proportion of hypertensive patients with controlled BP between high and low-income settings. Understanding the reasons behind variation is key to improving hypertension control levels worldwide and achieving the ambitious United Nations Sustainable Development Goal of universal health coverage by 2030 [3, 4, 12].

Although clinical guidelines are widely deemed as important in healthcare, there is only a small body of evidence investigating clinicians’ perceptions and use of guidelines in hypertension management. Studies at a national level have identified barriers to the implementation of hypertension guidelines, particularly in LMIC primary care settings, citing poor adherence and awareness of hypertension guidelines as a major area of concern [4, 7, 13, 14]. Chalmers argues that the main purpose of guidelines is to provide an authoritative and scientifically supported ‘reference point that codifies the accepted changes in practice in an area that is constantly evolving’ [15] and guidelines have become ubiquitous at local, national and international levels, largely to imbue evolving scientific evidence into routine clinical practice [13, 16]. However, the preferential use of one guideline over another has potentially far-reaching consequences, particularly when considering the multiplicity and variation within guidelines [15, 17]. For example, the lowering of the diagnostic threshold for hypertension to 130/80 mmHg in the latest American College of Cardiology and American Heart Association (ACC/AHA) guidelines [18], if adopted in the setting of an LMIC, may create a burden of need in the absence of clear evidence for conferring benefit to low-risk patients [13, 19].

Key issues relating specifically to hypertension guidelines are beginning to be explored. Firstly, international guidelines may be limited in their original form to be tailored to the local settings of LMICs due to the lack of available evidence relating to local populations and awareness of local context [3, 20]. Diagnostics, such as home BP monitoring or certain recommended medications, are often unavailable and unaffordable [3, 9]. There are subsequent impacts on practitioner beliefs as it has been reported that clinicians in these LMICs have less trust in local guidelines’ reliability compared to international guidelines, compounded by the fact that such guidelines are infrequently updated [9, 16]. Other important issues impacting use of guidelines lie within the healthcare system, health infrastructure, or relate to patient factors. Time pressures of primary care appointments, weak primary care health infrastructure, physician inertia and additional workload created by guideline recommendations have been described as barriers in both HICs and LMICs [20–22]. Patient factors, such as cultural acceptability of lifestyle interventions as well as low income households being unable to afford costly medication and healthy food also influence applicability of guidelines [13, 23]. Poor patient health literacy was identified as a significant barrier to BP control in a rural Rwandan district hospital study, echoed as a barrier in Argentina, urban Mongolia and within some HICs as well [7, 16, 22, 24–26].

Although factors influencing the use of hypertension guidelines have been reported, there is a significant underrepresentation of LMICs in the body of literature. This study aims to identify how hypertension guidelines are used by clinicians across a range of resource settings, healthcare settings and medical specialties. The paper explores the factors influencing clinician use of hypertension guidelines and distinguishes factors identified by clinicians in the context of preferential use of certain guidelines above others in their clinical practice, to better understand the disparities in hypertension control and variability in clinical practice.

## Methods

This qualitative study used in-depth semi-structured interviews with clinicians across a variety of resource settings to explore perceptions, beliefs, and experiences relating to the use of hypertension guidelines in their clinical practice. Participants were recruited through convenience sampling, using snowballing via emails to MMM national investigators who had previously consented to being contacted by the study team [27, 28]. The MMM campaign was initiated by the International Society of Hypertension (ISH) in 2017 and has run annually, including over 90 participating countries [11]. National investigators were asked to recommend healthcare workers in their countries who were available for interview between March and August 2020 and had given their consent to be contacted. Eligible English-speaking healthcare workers included doctors, nurses and community health workers who routinely managed hypertension, in both rural and urban locations, primary and secondary care, community health centres and hospitals, including both specialists and generalists. All clinicians identified by national investigators were medical doctors. Potential interviewees were emailed a participant information sheet (Additional file 1) and consent form. Written informed consent from all participants was obtained prior to interview commencement.

A semi-structured interview guide (Additional file 2) was created de-novo to facilitate the interviews and piloted by authors AKG, JC, NP and TB. Open-ended questions were utilised then further developed as the interview process unfolded to cater for different healthcare experiences and facilitate the extraction of broader insights of participants. From March to August 2020, seventeen interviews were conducted by AKG, a female medical student and BSc Global Health student at Imperial College London with formal training in qualitative interviewing methods and analysis. The interviewer was not previously known to the interviewees, but the rationale for the research and personal motivation was explained prior to interview. The initial two interviews were conducted jointly with TB, a male medical doctor and researcher at Imperial College London with prior training and experience in qualitative research. Each interview lasted between 40 and 60 min, in the form of a Skype video call or telephone call, depending on participant preference. Each was audio-recorded, transcribed verbatim and field notes on non-verbal cues for video calls were included in the transcripts. No repeat interviews were required and transcripts were not returned to participants. Data collection was stopped once thematic saturation was perceived to have been reached.

The interviews were coded and analysed using reflexive thematic analysis, to identify patterns in the data [29]. A reflexive approach was chosen given the flexibility of application without the requirement for a specific theoretical framework [30]. The seventeen transcribed interviews were entered into NVivo 12 and coded using a semantic approach, encompassing the essence of participant’s speech in the form of brief phrases or singular words [31]. A predominantly inductive and data-driven approach was used in generating codes and themes, however, a deductive approach was also applied to code only those concepts specifically relating to the research question [29, 30]. Data extracts were thus coded once, more than once or not at all depending on relevance. AKG was the sole coder of transcripts and systematically coded them based on the order of interview. An initial list of codes was created from the first coded transcript and expanded as more transcripts were coded. Uncertainties in coding were discussed with TB and JC.

Codes were then sorted and grouped with similar codes and groupings between codes considered to generate initial themes, which were refined through an iterative approach [29]. A predominantly inductive approach was used in theme generation, but a deductive approach was applied to consider themes at three socio-ecological levels: that of the practitioner, their interactions with patients, and the institutional and system settings. With regards to epistemological assumptions, knowledge formation was perceived through the lens of a realist framework which guided the thematic analysis and interpretation [29]. Levels and themes were discussed by AKG and TB, refining the results and key findings. Participants did not provide any direct feedback on the findings. The size of healthcare setting was defined according to Organisation for Economic Co-operation and Development definition of urban population by city size where possible [32]. Countries of participants and their associated national income levels were classified in accordance with The World Bank [33].

## Results

Interviews were conducted with seventeen clinicians in fourteen countries, all of whom were medical doctors. No participants dropped out of the study at any stage. The characteristics of the study participants are displayed in Table 1. Two clinicians worked in LICs, ten were from middle-income countries (MICs), and five worked in HICs. Additionally, seven doctors had experience in creating hypertension guidelines.

**Table 1:** Participant characteristics

Characteristic	Number (%)
**Sex**	
Female	5 (29.4)
Male	12 (70.6)
**Countries (*n*=14)**	
Azerbaijan	1 (5.9)
China	1 (5.9)
Hungary	2 (11.8)
India	2 (11.8)
Jamaica	1 (5.9)
Kyrgyz Republic	1 (5.9)
Nepal	2 (11.8)
Nigeria	1 (5.9)
South Africa	1 (5.9)
Sudan	1 (5.9)
Uganda	1 (5.9)
United Arab Emirates	1 (5.9)
United Kingdom	1 (5.9)
United States	1 (5.9)
**National income level of country**	
Low	2 (14.3%)
Lower middle	4 (28.6%)
Upper middle	5 (28.6%)
High	6 (28.6%)
**Years of healthcare experience**	
<10	7 (41.2)
10-19	3 (17.6)
20-29	1 (5.9)
30-39	4 (23.5)
Not recorded	2 (11.8)
**Clinical specialism**	
Cardiologist	6 (35.3)
Clinical physiologist	1 (5.9)
General practitioner (GP)	5 (29.4)
General practitioner trainee	2 (11.8)
Internal medicine trainee	2 (11.8)
Nephrologist	1 (5.9)
**Healthcare setting**	
Hospital	12 (70.6)
Rural	2 (11.8)
Medium-sized town	2 (11.8)
Urban	4 (23.5)
Capital city	4 (23.5)
General practice	5 (29.4)
Rural	1 (5.9)
Urban	1 (5.9)
Capital city	3 (17.6)

**Fig. 1:**
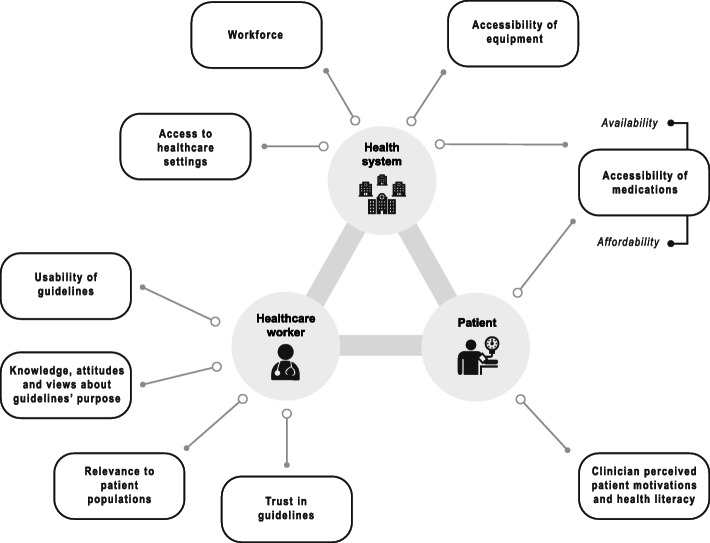
Thematic representation of the factors influencing clinician use of hypertension guidelines

The three main areas identified corresponded to the levels at which influencing factors were perceived to be operating (Fig. 1). These levels were: (1) Healthcare worker, (2) Healthcare worker interactions with patients, (3) Health system influences.

### Healthcare worker

Clinicians regarded generic guideline features such as usability and trust in the evidence-base that informs recommendations as important influences. Themes specific to clinicians such as knowledge and beliefs, and relevance to patient populations were deemed important.

#### Usability of guidelines

Guideline usability was identified by all doctors as an important influencing factor. Many doctors expressed the importance of format, with a preference for brief, simplified guidelines. The ease of use was synonymous with their ability to quickly read the guideline and refer back to it, as most doctors reported having insufficient time to read through long, complicated texts due to heavy workloads and the plethora of guidelines that exist for different medical conditions. This was felt by both General Practitioners (GPs) and hospital specialists across all national income settings.

The majority of participants expressed that they had simplified and shortened hypertension guidelines to make them easier to understand and use by other healthcare professionals regularly managing hypertensive patients within their department. Language of guidelines was a barrier identified by a few clinicians from LMICs only, especially in rural settings where English was often not spoken by clinicians. They recognised this as a barrier for non-English speaking doctors’ ability to access international guidelines, sometimes mitigated through its translation into the national language.

#### Trust in guidelines

Most participants expressed a preference for international guidelines over national or local guidelines due to having a greater level of trust in how they were formed and their subsequent reliability. This appeared to hinge on the high value placed on evidence-based medicine and rigorous scientific trials, often from HICs, that formed international guideline recommendations. The importance of strength of evidence was manifested by some participants’ (all of whom were hospital specialists from HICs) disapproval of the 2017 ACC/AHA guideline’s lowered threshold for diagnosing hypertension, despite its use of HIC level data:

“We think that the American guideline was a bit too quickly released and it was not really adjusted for the GP’s everyday practice… so we thought that it was wiser to stay with the European guideline.” (GP from a European HIC).

It was also perceived as having too small an evidence-base to substantiate a diagnostic change of such significance, as explained by a HIC cardiologist:

"We think it was a little bit too slim, or a little bit too early to follow this based on only one trial. We need many more trials before we go for this severe change."

Increased trust in international guidelines was contributed to by how regularly they were updated, which most participants recognised as crucial to maintaining current, up-to-date clinical practice. All participants from LICs and lower MICs recognised that their national guidelines were often outdated or non-existent.

#### Knowledge, attitudes and views about guidelines’ purpose

Clinicians’ views about what role guidelines played in clinical practice influenced their use. They were recognised as reference tools for standardising clinical practice as many doctors were aware of variation in hypertension management within their country. Guidelines were seen to positively contribute to consistent patient care across GP and hospital settings, as well as forming a useful reference point for their personal practice.

“The challenge we have is that when we have physicians, new graduates who are coming out to the field, they sometimes have challenges if they meet diseases they have never managed before… if you are looking at some references, it also gives you confidence that you are doing the right thing.” (GP from an African MIC).

A handful of participants said they followed national guidelines because they had been adapted from international or other national guidelines by a governmental health body or national hypertension society. Therefore, they felt that they had increased appropriateness for their local setting. Many GPs across income settings referred to the idea of growing familiarity with guidelines developing through repeated use over time. This meant they only explicitly referred to guidelines in cases which were less routine for them or that they were less clinically familiar with, but implied implicit use of the guideline through internalising its content. A GP from an upper MIC explained:

“Over time, there are guidelines you have become comfortable with, the medications that work, so it kind of becomes second nature…so, the only time you would need to go back to the guidelines is if you’re having difficulty controlling the blood pressure.”

#### Relevance to patient populations

Almost all doctors found difficulty in applying guidelines to certain patient subgroups. They identified the elderly, very young, patients with multiple comorbidities, polypharmacy, resistant hypertension, hypertensive emergencies, rare diseases, patients presenting with end-organ damage, non-compliant patients and specific ethnic populations, such as South Asian and Afro-Caribbean, as very challenging. Many doctors reported seeking alternative information sources to aid decision-making, including asking senior colleagues, reading published papers in medical journals, and searching the internet for similar cases. Some clinicians emphasised the importance of acting in patients’ best interests and felt that holistic patient management was crucial in such circumstances. Ethnicity-specific challenges arose in terms of the applicability of hypertension risk scores and the choice of medication at each treatment step. Many clinicians believed insufficient trial level data for certain ethnic subgroups meant guidelines could not be made as relevant to their particular population.

### Healthcare worker interactions with patients

Clinicians recognised a disparity in the lifestyle recommendations provided by hypertension guidelines and their perception of patients’ willingness or ability to enact lifestyle recommendations. These were discussed in terms of patient motivations and health literacy. Affordability of treatment was inextricably bound to the structure and provision of the health system, limiting guideline use in certain patient circumstances.

#### Clinician perceived patient motivations and health literacy

Almost all doctors across all resource settings expressed difficulties in engaging patients with lifestyle advice provided by hypertension guidelines, often framed as patients’ unwillingness to attempt or maintain lifestyle improvements, or an inadequate understanding of hypertension, its risk factors and sequelae.

Doctors’ perception of patients as unwilling or resistant to lifestyle advice represented a tension between doctors and patients in the context of guideline recommendations. Although most doctors felt that they were able to communicate the lifestyle recommendations provided by guidelines to their patients, they postulated various reasons as to why advice was challenging to follow, most of which implied fault of the patient: low motivation and commitment to making healthier choices and monitoring BP at home, apathy, poor acceptance of diagnosis, and reluctance to diverge from cultural norms. However, some clinicians alluded to the influence of wider determinants of health, such as education, rather than the fault the individual.

Most clinicians felt they regularly encountered patients who were misinformed about hypertension treatment’s preventive rather than curative nature, and had poor understanding of the importance of reducing risk factors. The perceived insufficient knowledge of hypertension was often referred to by clinicians as ‘poor health literacy’ which the media, as well as socio-economic factors such as educational attainment, contributed to. Low educational attainment contributing to poor patient health literacy was expressed by clinicians from LMICs or those working in rural settings, often resulting in late presentation to health services.

The consequences of fundamental gaps in patients’ knowledge about hypertension affected clinicians’ use of guidelines in two main ways. Some clinicians expressed that applying guidelines became a lengthy process when patients were uninformed or misinformed about the condition or the implications of treatment as they had to spend time addressing these. Secondly, a handful of clinicians in lower MICs decided to create initiatives that addressed the barriers as a prior step to the use of hypertension guideline lifestyle recommendations. A South African GP outlined a personal initiative to address lifestyle modifications that acknowledged the social and cultural context of their clinic’s local setting:

We’ve created support groups for uncontrolled hypertensive and obese people where they come together every Thursday…I put together an approach that’s based on the township reality, the reality of the people who live there. So they kind of support each other and help each other lose weight and give each other advice.

#### Accessibility of medications: availability and affordability

Patients’ ability to afford the prescribed antihypertensives was an important influencing factor identified in most resource settings. Some clinicians stated expensive health insurance as limiting patients’ compliance with treatment and felt that they could not achieve best practice, deviating from guideline recommendations. A consequence of cost constraints, raised by a cardiologist from a MIC, was that some patients bought the cheapest version of a medication, which “maybe doesn’t work well because it is very cheap and is not so effective as a branded drug or a generic with a higher price.” Therefore, the recommendation of certain drugs in the guideline can be viewed as limiting its usefulness within clinicians’ settings.

### Health system influences

Accessibility of resources relating to the health system was discussed in terms of barriers or facilitators to the use of hypertension guidelines. Common factors elucidated were accessibility of treatment (including availability of equipment and antihypertensives), human resources, and access to healthcare settings.

#### Accessibility of equipment

Many healthcare workers explained that their ability to follow hypertension guidelines for diagnosis and management was heavily influenced by the equipment and investigations available in their healthcare setting. Some doctors working in LMICs reported low availability of diagnostic and monitoring tools such as home BP monitoring machines. This sometimes affected guideline use as doctors felt that they could not adequately monitor patients’ hypertension, leading to a variety of consequences such as delayed diagnosis and late patient presentation to health services with complications.

A number of clinicians from lower MICs expressed difficulties with diagnosing certain comorbid conditions that were accounted for in their hypertension guideline treatment algorithms, due to a scarcity of diagnostic resources in their healthcare setting. A doctor working in a rural hospital in a lower MIC expressed his inability to follow the chronology of antihypertensive drug classes for CKD patients:

"In the guidelines it has been mentioned that for patients with chronic kidney disease we have to follow a different [treatment] pattern. But where I’m working, I can’t generally identify the individuals with chronic kidney disease. I can’t separate them."

#### Accessibility of medications: availability and affordability

The availability and affordability of medications recommended by guidelines was deemed by most clinicians to be influencing what drugs they could prescribe, with availability of medications in pharmacies and the affordability of antihypertensives the main contributors to accessibility. Accessibility of medications was discussed at both the level of the healthcare system and the clinician relationship with patients, due to patients’ ability to afford medication relying on health system infrastructure and insurance policies in addition to their own finances. Most doctors reported no issues with accessing drug classes recommended by guidelines, but some clinicians in both HICs and LMICs expressed issues with their hospital’s restocking of drugs and unavailability of drugs considered less cost-effective.

“In our set up amlodipine is much more expensive than nifedipine. So, most of the time we are tempted to prescribe nifedipine because it’s cheaply available and more affordable for the patient.” (GP in a LIC).

The majority of clinicians from LMICs expressed the view that insurance policies dictated whether patients could afford many medications, also feeling limited by the range of antihypertensives that were covered by governmental free healthcare. A GP from an upper MIC reflected upon the factors influencing the medications prescribed for her patients: “The choice of medication usually would be dependent on which setting the patient goes to and what the patient can afford.” A few clinicians in European HICs found the European Society of Cardiology/European Society of Hypertension (ESC/ESH) recommendation for initiating single-pill combination therapy challenging due to low availability in pharmacies, which they felt was a result of its shorter shelf-life and lower profitability for pharmacies.

#### Workforce

A problem identified by hospital specialists practicing in LMICs was the limited number of specialist doctors in their settings. A consequence was that they had limited time to counsel patients and could not ensure close adherence to the guidelines as a result. An additional difficulty was the shortage of senior colleagues in rural hospitals, expressed by a few clinicians in both LMICs and HICs. Doctors felt they could not easily seek advice when faced with complex patients that hypertension guidelines did not cater for. A nephrologist from a lower MIC reflected on the challenges:

"Well, it’s quite difficult to use guidelines when you are working in a rural centre. Because number one, the best practice is not there…so the [clinical] experience will not be there. And unfortunately, you don’t have any senior colleagues in these areas."

#### Access to healthcare settings

Healthcare workers across all national income settings reported distance to GP surgeries or hospitals, the time taken to attend doctors’ appointments, and the cost of travel for patients as factors influencing patient compliance with treatment. Some clinicians regarded poor national healthcare infrastructure as the cause, especially in rural or semi-rural areas. A GP from a European HIC explained how far rural patients’ far travel to reach specialist centres placed greater pressure on rural GPs:

"How far a doctor is, how far they have to travel to get an ultrasound or to get a cardiology specialist. In the countryside it’s much, much worse and there’s a higher challenge for GPs to solve their problems in general."

## Discussion

This qualitative study investigating clinicians’ views of factors influencing their use of hypertension guidelines was the first of its kind to sample practitioners across a wide geographical spread and to include countries representing all levels of national income, including two LICs, eight MICs and four HICs. Facilitators and barriers to guideline use were identified at three levels of influence: healthcare worker, healthcare worker relationships with patients and with the health system. Key factors common to all resource settings were accessibility of equipment, accessibility of medications for patients, guideline usability and familiarity, trust in the evidence-base used to form guidelines, relevance to patient subgroups, clinician perceived patient motivations and health literacy. In the context of differences in hypertension control and awareness between countries, the results represent the perspectives of an extensive range of participants from various national income settings, primary and secondary care, and rural and urban locations. However, the barriers to guideline implementation elucidated by clinicians should not be interpreted as the sole explanations for why BP control may be low, but rather as highlighting circumstances requiring further and more specific investigation with regards to guideline use.

### Healthcare worker level influences

For clinicians, usability was the key influencing factor and limited time to read guidelines in a clinical setting appeared to be a common barrier. The Reassessing European Attitudes about Cardiovascular Treatment survey [34] highlighted accessible, simple guideline format as one of three major attributes of ‘a useful clinical guideline’ and the negative implications of poor guideline usability identified in national level primary care studies in MICs also support this finding [20, 22]. Many clinicians stated having higher trust in international guidelines rather than national guidelines, mostly due to their regular updating and evidence-based recommendations. This echoes the finding of a qualitative study exploring healthcare professionals’ views of a Malaysian hypertension guideline which emphasised that ‘issues inherent within the guidelines that may lead to scepticism by the healthcare providers (such as outdated and unreliable information)’ must be addressed prior to any policy changes [20] An observational study of GPs in the Netherlands not only found that evidence-based guideline recommendations were used more by GPs than recommendations without such basis, but that ‘an explicit description’ of the evidence in guidelines conferred greater adherence [35].

Although issues stemming from international or other national guideline use in LMICs have started to be discussed in the literature, clinicians from all resource settings in this study recalled difficulties applying international or national guidelines to certain patient subgroups [3, 20]. A systematic review of guidelines in LMICs discussed the process of adapting existing HIC guidelines into LMIC guidelines as ignoring ‘contextually relevant locally-derived evidence’ which may partially explain these difficulties [7]. It is important to note that this constitutes one of a variety of reasons, operating at different strata of healthcare, why guidelines may be difficult to implement in LMICs. Participants explained seeking alternative information sources to make clinical decisions, using the best available evidence and personal or senior colleague experience. Many clinicians expressed Afro-Caribbean and South Asian ethnicities as difficult to apply evidence-based treatment recommendations to. They commonly believed that improving trial level data for optimal antihypertensive drug therapy in these groups was crucial to creating more applicable guideline recommendations.

### Healthcare worker interactions with patients

Clinicians mostly discussed practitioner-patient level influences as barriers, namely their perception of patients’ views of lifestyle information as well as general misinformation or inadequate knowledge about hypertension. Although there is a distinction between provision of lifestyle advice recommended by guidelines and patient autonomy, frictions between doctors and patients seemed to exist in the domain of lifestyle recommendations. This was not expressly mentioned by clinicians, but a dissonance was evident in the content of the interviews. This may have impacted how doctors conveyed lifestyle recommendations to their patients, but it requires further investigation to the impacts of the finding. Although participants could not definitively express patients’ own motivations, it is noteworthy that similar barriers have been noted previously across vastly different healthcare settings [8, 24, 36]. Future qualitative research interviewing patients could aid understanding of their perception of barriers to concordance with recommended treatment, which may differ from the views of healthcare professionals.

To overcome the perceived barrier of poor health literacy, clinicians felt they had to bridge the gap between patient understanding of treatment implied by guideline recommendations and the reality of patient health knowledge, which was felt to be a complex and challenging task. However, doctors may have varied understanding of ‘health literacy’ and its impacts on hypertension management. Existing research of varying scope and methodology provides compelling evidence that low levels of patient health literacy with respect to chronic illness limit treatment compliance, especially lifestyle changes [4, 8, 24, 37].

### Health system influences

The study identified limited resources at the health system level across national income settings, including difficulties accessing diagnostics and medication affordability for patients. In LMICs, clinicians described specific barriers such as unaffordable medication cost, insufficient numbers of healthcare workers and senior consultants, and low availability of BP monitors as impeding their use of guidelines. The factors identified in LMICs have been described by previous studies, but differences in participants’ experiences and their local settings mean that caution should be exercised in comparing contexts [23, 24, 38] Mills et al. suggested an ‘urgent need to identify innovative strategies to overcome these barriers’ [4] and Chow et al. indicated alternative approaches to addressing these LMIC specific barriers, such as a shift of hypertension diagnosis from medical to non-medical healthcare workers [38]. Although health system level influences are location-specific, heterogeneous and heavily influenced by policy, international guidelines providing wider treatment options to include ‘essential’ and ‘optimal’ choices that allow a minimum standard of care, as exemplified by the 2020 ISH guideline, may facilitate greater guideline adherence in LMICs [39].

### Strengths and limitations

To our knowledge, this is the first study investigating the influences on hypertension guideline use by clinicians across a range of healthcare and national income settings. The use of convenience sampling could be viewed as a limitation of the study due to potential selection bias. As a result, the sampling strategy may make the findings here lack generalisability, as influencing factors and barriers can differ by resource setting, but it still crucially permits transferability of the findings which could impact future research in this area [40, 41]. Although the recruitment strategy and inclusion criteria initially aimed to include a variety of clinical staff such as nurses and community health workers, only medical doctors were interviewed. However, a variety of perspectives from different medical specialties, healthcare settings and resource settings were explored. Although the overall sample was small and included few LIC participants, recruitment was stopped once no new themes were identified. Local context is acknowledged as important to sample representativeness, but elaboration in the interviews of country-specific contexts contributing to participants’ experiences would have been beyond the study’s scope [42]. Future studies of a similar nature would benefit from purposive sampling techniques, to incorporate more participants from LMICs and enabling comparison across resource settings and within different LMIC contexts where hypertension is significantly under controlled [43].

Seven participants had experience in creating hypertension guidelines, due in part to the recruitment through national investigators of MMM, which could have impacted the results due to greater insight into guideline creation and implementation. Furthermore, these participants may have been less likely to identify barriers to guideline use as their involvement with creating the guideline could positively influence their perception of its utility. In addition, only English-speaking participants were included which may limit the generalisability to non-English-speaking settings.

### Recommendations for practice

Hypertension guidelines, at international, national and local levels, should be evidence-based and regularly reviewed and updated to increase reliability of recommendations and enable translation of new evidence into clinical practice. Clear formatting and appearance of guidelines should be developed, user-tested and employed in guideline creation to ease use by time-pressured clinicians. Simplified summarised sections of guideline recommendations may also enhance usability by clinicians. The recommendations to improve ease of guideline use may be applicable to the innovation of clinical guidelines on a wider scale. Greater acknowledgement in hypertension guidelines of local demographics such as ethnic variation and variation in social and economic contexts could enable more tailored and specific approaches to the provision of lifestyle advice in different contexts which may improve clinician adherence to the guideline. Further development of local guidelines, perhaps based on international and national guidelines, may facilitate this.

### Recommendations for research

Expanding participant representation in qualitative studies to include patients with hypertension would aid our understanding of the barriers to guideline use, as perceived by patients themselves. Patient perceived factors may differ from those perceived by clinicians and might further elucidate the tensions identified in our study by clinicians in the doctor-patient relationship when recommending lifestyle changes. These findings may enhance the tailoring of certain guideline recommendations that are poorly implemented, including lifestyle and dietary advice, to patients. Further studies should be inclusive of other healthcare professionals who regularly manage hypertension, including nurses and community health workers. Despite poorer hypertension awareness and control rates in LMICs compared to HICs, there is a scarcity of qualitative studies conducted in LMICs. Cabana et al.’s review of barriers to guideline implementation importantly notes barriers beyond lack of knowledge or awareness. Physicians’ lack of agreement with guidelines, such as the belief that guidelines may be oversimplified, the benefits to patients not being worth the risk, or that they would reduce physician autonomy, were recognised barriers to guideline implementation in general. Other barriers that mean guidelines may not be easily used or followed, such as environmental difficulties or lack of self-efficacy, may have particular relevance to LMIC settings [40]. Research addressing the paucity of evidence for implementation of guidelines specifically in LMIC settings could inform LMIC-specific guideline recommendations in the future, incorporating greater consideration or acknowledgement of the socio-economic and cultural factors that influence the use of guidelines.

## Conclusions

This study adds a global perspective on prior studies investigating clinician perspectives on hypertension guideline use. Factors pertaining to the healthcare worker, and their interactions with patients and the health system are important influencers of the use of hypertension guidelines by clinicians across all national income settings investigated. Facilitators and barriers of guideline use, which favour preferential use of certain guidelines over others, were explored. Future qualitative research incorporating wider stakeholder participation, including patients, and focused particularly in LMICs is necessary to improve guideline innovation and applicability to different resource settings.

## Supplementary Information

**Additional file 1.**

**Additional file 2.**

## Data Availability

Transcripts of interviews are retained by the study team, but are not publicly available or available on request due to the potential for interviews to disclose potentially identifiable information.

## Abbreviations

ACC/AHA: American College of Cardiology/ American Heart Association
BP: Blood pressure
CKD: Chronic kidney disease
ESC/ESH: European Society of Cardiology/ European Society of Hypertension
GP: General practitioner
HIC: High-income country
ISH: International Society of Hypertension
LIC: Low-income country
LMIC: Low- and middle-income countries
MIC: Middle-income country
MMM: May Measurement Month
