# Supplementary material for: The factors influencing clinician use of hypertension guidelines in different resource settings: a qualitative study investigating clinicians’ perspectives and experiences
Source: BMC Health Serv Res. 2021 Aug 3;21:767. doi: 10.1186/s12913-021-06782-w (PMC8336017; doi:10.1186/s12913-021-06782-w)
Supplement: Supplementary file 1 — Additional file 1. [file 12913_2021_6782_MOESM1_ESM.pdf]

The factors influencing clinician use of hypertension guidelines in different resource settings: a qualitative study investigating clinicians' perspectives and experiences.

Authors: Amelia Kataria Golestaneh[1], Jonathan M Clarke[2], Nicholas Appelbaum[3], Carmen Rodriguez Gonzalvez[3], Arun P Jose[4], Richu Philip[1], Neil Poulter[5], Thomas Beaney[1]

[1] Department of Primary Care and Public Health, Imperial College London, London, United Kingdom

[2] Centre for Mathematics of Precision Healthcare, Department of Mathematics, Imperial College London, London, United Kingdom

[3] Institute of Global Health Innovation, Imperial College London, London, United Kingdom

[4] Centre for Chronic Conditions and Injuries, Public Health Foundation of India, New Delhi, India

[5] Imperial Clinical Trials Unit, School of Public Health, Imperial College London, London, United Kingdom

Corresponding author:

Dr Thomas Beaney

Department of Primary Care and Public Health, St Dunstan's Road, London, W6 8RP, United Kingdom

Email: Thomas.beaney@imperial.ac.uk

Participant Information Sheet

A INTRODUCTION

Hypertension is the single largest contributor to the global burden of disease, with its prevalence expected to rise, particularly in low and lower-middle income countries. Agreed standards on who should be diagnosed with hypertension, and how they should be managed vary significantly between guidelines. Some guidelines may be more applicable to a particular country or setting, while others may be limited by the available evidence relevant to their population.

Clinical guidelines are commonly used, at local, national and international level. Yet despite this, relatively little research has explored how clinicians decide *which* guidelines to use in a given case, and what factors influence which parts of guidelines they use and why. This study aims to identify how clinician decisions on guideline use are influenced.

B. PROCEDURES

A qualitative study of factors influencing clinician use of hypertension guidelines in different resource settings

Principal Investigator: Dr Thomas Beaney
Co-Investigators: Amelia Kataria Golestaneh, Dr Jonathan Clarke, Dr Nicholas Appelbaum, Dr Carmen Rodriguez Gonzalvez, Professor Neil Poulter

You are being invited to take part in this research study. Before you decide it is important for you to understand why the research is being done and what it will involve. Please take time to read the following information carefully and discuss it with others if you wish.

Part 1 tells you the purpose of this study and what will happen to you if you take part.
Part 2 gives you more detailed information about the conduct of the study

Ask us if there is anything that is not clear or if you would like more information. Take time to decide whether or not you wish to take part.

Thank you for taking the time to consider this.

- **What is the purpose of the study?**

This study aims to identify the factors influencing use of guidelines amongst clinicians. The focus is on use of guidelines in hypertension, but the findings will be applicable more widely to understanding how guidelines are used.

Although guidelines are used all over the world, and at local, national and international level, little research has been carried out into what determines which guidelines are used, and how they are used.

This study will be conducted by Amelia Kataria Golestaneh, a BSc student in Global Health at Imperial College, supervised by the Principal Investigator and co-Investigators.

- Why have I been invited?

You have been invited to participate as a collaborator of the May Measurement Month (MMM) campaign, or on the recommendation of the national leader for MMM in your country, as a healthcare professional who routinely diagnoses and manages patients with hypertension. We are aiming to recruit between 30 and 40 clinicians to share their valuable insight into how they use clinical guidelines, and what makes these guidelines effective.

- Do I have to take part?

It is up to you to decide whether or not to take part. If you do decide to take part you will be given this information sheet to keep and be asked to sign a consent form. If you decide to take part you are still free to withdraw at any time and without giving a reason, by informing any of the investigators.

- What will happen to me if I take part?

Involvement in this study will take place as a single Skype interview, lasting between 30 and 60 minutes at a time convenient to you. If a stable internet connection is unavailable, then an interview via telephone call can instead be arranged. The interview will take place via a semi-structured methodology conducted by Amelia Kataria Golestaneh, BSc student in Global Health at Imperial College. This will focus on how you use guidelines (if at all) in the diagnosis and management of hypertension and how guidelines could be useful to you. This audio from the interview will be recorded and then transcribed. Once transcribed, the recording will be deleted. Responses will be anonymised for the purposes of the research, and only a limited amount of information on you will be recorded for the purposes of analysing the results (including job title, healthcare setting and country).

- **What do I have to do?**

The interview will be conducted over Skype or telephone call in March - early April 2020. Unfortunately, we do not have the funds to provide translation during the interviews, and so a conversational level of English is required.

There are no other specific requirements. You are welcome to conduct the interview in any appropriate setting, and we can be flexible in terms of timing of the interview to work around your schedule.

- **What are the possible disadvantages and risks of taking part?**

We do not expect there to be any disadvantages to taking part in this study. If at any time during the interview, you feel uncomfortable, you have the right to withdraw by letting the interviewer and/or one of the investigators know. Any responses collected by that point will be securely erased. Once the audio has been transcribed, and thematic analysis has begun, it will not be possible to withdraw. However, all responses will be anonymised, although a limited amount of information on you will be included (job title, healthcare setting and country).

- **What are the possible benefits of taking part?**

There are no direct benefits to you as a participant in taking part in this study. However, we hope that the results obtained will be of use in the design of clinical guidelines in the future, and in making these more suitable for different healthcare settings, which may have an indirect benefit for you, your colleagues and patients.

- **What if something goes wrong?**

If you are harmed by taking part in this research project, there are no special compensation arrangements. If you are harmed due to someone's negligence, then you may have grounds for a legal action. Regardless of this, if you wish to complain, or have any concerns about any aspect of the way you have been treated during the course of this study then you should immediately inform the Principal Investigator (Dr Thomas Beaney, email: thomas.beaney@imperial.ac.uk). If you are still not satisfied with the response, you may contact the Imperial Joint Research Compliance Office.

The risk of any adverse events is low, but any serious adverse events will be reported to the Ethics and Research Governance Coordinator at Imperial College London.

Transparency notice

- Will my taking part in this study be kept confidential?

Audio recordings of interviews will be stored confidentially in line with the General Data Protection Regulations and available only to the study team. Once transcribed (within one month of the interviews), the recordings will be securely erased. Additional information collected on you as a participant will include your job title, years of healthcare experience, location of health care setting and country of work. Your name and contact details will be stored securely on Imperial college servers in a password protected file with a unique identifier linked to the interviews. Your name and contact details will not be included in any study results but your general job title, healthcare setting and country may be reported and has the potential to be identifiable.

HOW WILL WE USE INFORMATION ABOUT YOU?

Imperial College London is the sponsor for this study and will act as the data controller for this study. This means that we are responsible for looking after your information and using it properly. Imperial College London will keep your personal data for:

- 10 years after the study has finished in relation to data subject consent forms.
- 10 years after the study has completed in relation to primary research data.

We will need to use information from you for this research project.
This information will include your name, contact details, job title, years of healthcare experience, location of health care setting and country of work. People will use this information to do the research or to check your records to make sure that the research is being done properly.
People who do not need to know who you are will not be able to see your name or contact details. Your data will have a code number instead. We will keep all information about you safe and secure. Once we have finished the study, we will keep some of the data so we can check the results. We will write our reports in a way that no-one can work out that you took part in the study.

LEGAL BASIS

As a university we use personally-identifiable information to conduct research to improve health, care and services. As a publicly-funded organisation, we have to ensure that it is in

the public interest when we use personally-identifiable information from people who have agreed to take part in research. This means that when you agree to take part in a research study, we will use your data in the ways needed to conduct and analyse the research study. Health and care research should serve the public interest, which means that we have to demonstrate that our research serves the interests of society as a whole. We do this by following the UK Policy Framework for Health and Social Care Research

INTERNATIONAL TRANSFERS

There may be a requirement to transfer information to countries outside the European Economic Area (for example, to a research partner). Where this information contains your personal data, Imperial College London will ensure that it is transferred in accordance with data protection legislation. If the data is transferred to a country which is not subject to a European Commission (**EC**) adequacy decision in respect of its data protection standards, Imperial College London will enter into a data sharing agreement with the recipient organisation that incorporates EC approved standard contractual clauses that safeguard how your personal data is processed.

SHARING YOUR INFORMATION WITH OTHERS

For the purposes referred to in this privacy notice and relying on the bases for processing as set out above, we will share your personal data with certain third parties.

- Other College employees, agents, contractors and service providers (for example, suppliers of printing and mailing services, email communication services or web services, or suppliers who help us carry out any of the activities described above). Our third -party service providers are required to enter into data processing agreements with us. We only permit them to process your personal data for specified purposes and in accordance with our policies.

WHAT ARE YOUR CHOICES ABOUT HOW YOUR INFORMATION IS USED?

You can stop being part of the study at any time, without giving a reason, but we will keep information about you that we already have.

- We need to manage your records in specific ways for the research to be reliable. This means that we won’t be able to let you see or change the data we hold about you.

WHERE CAN YOU FIND OUT MORE ABOUT HOW YOUR INFORMATION IS USED

You can find out more about how we use your information

- by asking one of the research team
- by sending an email to the Principal Investigator: thomas.beaney@imperial.ac.uk

COMPLAINT

If you wish to raise a complaint on how we have handled your personal data, please contact Imperial College London's Data Protection Officer via email at dpo@imperial.ac.uk, via telephone on 020 7594 3502 and/or via post at Imperial College London, Data Protection Officer, Faculty Building Level 4, London SW7 2AZ.

If you are not satisfied with our response or believe we are processing your personal data in a way that is not lawful you can complain to the Information Commissioner's Office (ICO). The ICO does recommend that you seek to resolve matters with the data controller (us) first before involving the regulator.

- **What will happen to the results of the research study?**

The results of this study will be written up as a dissertation for a BSc in Global Health by the end of May 2020. Following this, the results will be submitted for publication in an academic journal. You will not be identified in any publication. If you would like to find out more about the proposed publication plan, or to request details following publication, please contact the Principal Investigator (thomas.beaney@imperial.ac.uk).

- **Who is organising and funding the research?**

This research is being organised and sponsored by Imperial College London. There is no funding for this project, beyond a small allocation of funds through Imperial College London to cover project consumables.

- **Who has reviewed the study?**

This study was given ethical approval by the Joint Research and Compliance Office (JRCO) and Professor Azeem Majeed, Head of the Department of Primary Care and Public Health, Imperial College London.

Contact for Further Information

For further information, please contact the Principal Investigator, Dr Thomas Beaney (thomas.beaney@imperial.ac.uk) or Professor Neil Poulter (n.poulter@imperial.ac.uk)

Thank you for taking the time to read and consider taking part in this study.
