# Supplementary material for: The factors influencing clinician use of hypertension guidelines in different resource settings: a qualitative study investigating clinicians’ perspectives and experiences
Source: BMC Health Serv Res. 2021 Aug 3;21:767. doi: 10.1186/s12913-021-06782-w (PMC8336017; doi:10.1186/s12913-021-06782-w)
Supplement: Supplementary file 2 — Additional file 2. [file 12913_2021_6782_MOESM2_ESM.pdf]

The factors influencing clinician use of hypertension guidelines in different resource settings: a qualitative study investigating clinicians' perspectives and experiences.

Authors: Amelia Kataria Golestaneh[1], Jonathan M Clarke[2], Nicholas Appelbaum[3], Carmen Rodriguez Gonzalvez[3], Arun P Jose[4], Richu Philip[1], Neil Poulter[5], Thomas Beaney[1]

[1] Department of Primary Care and Public Health, Imperial College London, London, United Kingdom

[2] Centre for Mathematics of Precision Healthcare, Department of Mathematics, Imperial College London, London, United Kingdom

[3] Institute of Global Health Innovation, Imperial College London, London, United Kingdom

[4] Centre for Chronic Conditions and Injuries, Public Health Foundation of India, New Delhi, India

[5] Imperial Clinical Trials Unit, School of Public Health, Imperial College London, London, United Kingdom

Corresponding author:

Dr Thomas Beaney

Department of Primary Care and Public Health, St Dunstan's Road, London, W6 8RP, United Kingdom

Email: Thomas.beaney@imperial.ac.uk

A qualitative study of factors influencing clinician use of hypertension guidelines in different resource settings

Semi-structured interview questions

"My name is Amelia, thank you again for agreeing to take part in this study. The interview will take between 30 and 60 minutes. Would it be alright with you if the videocall is recorded- it will be stored securely on a limited access Imperial server and deleted within 30 days of the interview. Please confirm that you have read the participant information sheet and signed the consent form, and that you understand the interview will be recorded and the dialogue will then be transcribed. If any question is unclear, please let me know and I can clarify. You are free to leave at any time during the interview without giving a reason."

Background to participant structured interview

"These are a few brief questions with yes or no answers"

1. Name
2. Job title
3. Healthcare setting including country
4. Years of healthcare experience (approximate)
5. Do you manage patients with hypertension?
6. How frequently?
7. Do you consider yourself a hypertension specialist?
    a. If yes: are patients referred to you? (How)
    b. If no: do you refer patients onwards to a specialist?

General opening questions

"The focus is not on clinical management, but more about how you use guidelines rather than the management recommended by the guidelines."

1. Do you use guidelines in the diagnosis and management of hypertension? Can you tell me more about your experiences
2. If yes, what do you use them for? (diagnosis, management, reference etc.)
   If not, do you use clinical guidelines for any other medical conditions?
   What information do you use?

Or:

3. What do you understand about guidelines in clinical practice?
4. How do you use guidelines in your day to day clinical practice?

Focused questions

Which?

- Which guideline or guidelines do you use? (local, national, international)
    - Note to self: clarify full name of guideline(s) and check if guidelines are written in multiple languages

Why?

Why do you use that guideline in particular?

When?

- Do you always use guidelines?
- In what circumstances would you use/ not use guidelines?
- Where would you seek information from in these situations?
- In what circumstances would you manage your patients differently from outlined in the guidelines?

How?

- How do you access guidelines?

Relevance?

- To what extent do you feel the guidelines you use are relevant to your setting?
  - E.g. rural vs urban, travel to services, acceptability of lifestyle advice differing
- Do you feel that there are sufficient guidelines to use for your local population?
- How could guidelines be made most relevant to you?

At this point in the interview, it would be useful to consider the following domains when discussing the question above:

Accessibility of guidelines:

- Language
- Ease of access to guidelines
  - different resource settings/ novelty of guidelines/ how implemented
- Ease of use

Applicability to the local population and healthcare beliefs:

- Relevance to patient population
- Relevance to subpopulations
  - ethnicity, age, comorbidities, polypharmacy
- Side effects / polypharmacy
- Acceptability for patients
  - cost, conflict with cultural beliefs, wellbeing, lifestyle, health literacy

Applicability to the resources of the health system:

- Cost (also who pays- patients or national/ private insurance)
- Accessibility of medications (e.g. bias of drug companies)
- Accessibility of diagnostics (how do you make your diagnosis of hypertension? Equipment accessibility, clinical vs home/ ambulatory)
- Accessibility of specialist referral
- Clinical pathways (e.g. secondary hypertension causes/ investigations)
- Time / time pressures / waiting lists

Doctor's beliefs and perceptions of local guidelines

- Local / National / International
- Trust
- Why trust certain guidelines more?

Local information sharing

- Are you aware of how nearby hospitals or clinics treat hypertensive patients? (peers & primary/ secondary care)
    a. If yes, is this helpful?
    b. If no, do you think it could be helpful and why?

Other: Have you worked in other resource settings and how/ what differed?

Closing questions

- Is there anything else you would like to discuss with regards to guidelines in clinical practice?
- Do you have any further questions?

"Thank you for taking the time to participate in this interview."
